# Boundedly rational expected utility theory

**DOI:** 10.1007/s11166-018-9293-3

**Published:** 2018-12-28

**Authors:** Daniel Navarro-Martinez, Graham Loomes, Andrea Isoni, David Butler, Larbi Alaoui

**Affiliations:** 1 Department of Economics and Business, Pompeu Fabra University, Ramon Trias Fargas 25-27, 08005 Barcelona, Spain; 2 Barcelona Graduate School of Economics, Barcelona, Spain; 3 Warwick Business School, University of Warwick, Coventry, CV4 7AL UK; 4 University of Cagliari, Cagliari, Italy; 5 Department of Accounting, Finance and Economics, Griffith Business School, Griffith University, Gold Coast, QLD Australia

**Keywords:** Expected utility, Bounded rationality, Deliberation, Probabilistic choice, Confidence, Response times, D03, D81

## Abstract

We build a satisficing model of choice under risk which embeds Expected Utility Theory (EUT) into a boundedly rational deliberation process. The decision maker accumulates evidence for and against alternative options by repeatedly sampling from her underlying set of EU preferences until the evidence favouring one option satisfies her desired level of confidence. Despite its EUT core, the model produces patterns of behaviour that violate standard EUT axioms, while at the same time capturing systematic relationships between choice probabilities, response times and confidence judgments, which are beyond the scope of theories that do not take deliberation into account.

**Electronic supplementary material:**

The online version of this article (10.1007/s11166-018-9293-3) contains supplementary material, which is available to authorized users.

## Introduction

Economics is often said to be the study of the allocation of scarce resources, of how human beings decide to combine their time and skills with physical resources to produce, distribute and consume. However, economic models may sometimes ignore the fact that arriving at decisions is itself an economic activity and that the hardware and software involved—that is, the human brain and its mental processes—are themselves subject to constraints. Herbert Simon emphasised this point in his 1978 Richard T. Ely lecture, in which he discussed the implications of attention being a scarce resource.^1^ In a world where there are many (often complex) choices to be made, spending time on any one decision entails an opportunity cost in terms of the potential fruits of other decisions that might have been considered instead. Being unable to devote unlimited time and attention to every decision they encounter, humans generally have to *satisfice* rather than optimise.

Simon bemoaned the lack of interest among economists in the processes that individuals use when deciding how to allocate their scarce mental resources, and he advocated “building a theory of procedural rationality to complement existing theories of substantive rationality”. He suggested that “some elements of such a theory can be borrowed from the neighboring disciplines of operations research, artificial intelligence, and cognitive psychology”, but noted that “an enormous job remains to be done to extend this work and to apply it to specifically economic problems” (Simon 1978, pp.14–15).

Although there have been some developments along these lines (e.g., Gilboa and Schmeidler 1995; Rubinstein 1988; Gigerenzer and Selten 2001), the decades that followed Simon’s lecture saw the mainstream modelling of individual decision making—especially with respect to choice under risk and uncertainty—take a different direction. Stimulated by experimental data that appeared to violate basic axioms of rational choice, a number of models appeared at the end of the 1970s and in the early 1980s that sought to provide behavioural alternatives to standard Expected Utility Theory (EUT)—see Starmer (2000) for a review. Typically, these were deterministic models that relaxed a particular axiom and/or incorporated various additional features—e.g., reference points, loss aversion, probability weighting, regret, disappointment—to try to account for certain regularities in observed decisions. While such models provided more elaborate descriptive theories of choice, little or no consideration was given to the mental constraints referred to by Simon. His invocation to build boundedly rational procedural models largely fell by the wayside in the field of risky decision making.

Thus we now have an impressive array of alternative deterministic models, each of which can claim to accommodate some (but not all) of the observed departures from EUT. However, these models have no intrinsic explanation for at least three other pervasive empirical regularities in the data, which may arise from features of the decision-making process: first, the probabilistic nature of most people’s decisions^2^; second, the systematic variability in the time it takes an individual to respond to different decision tasks of comparable complexity^3^; and third, the degree of confidence decision makers (DMs) express about their decisions.^4^

In this paper, we propose to explore the direction Simon advocated and investigate the potential for applying a boundedly rational deliberative process to the ‘industry standard’ model of decision making under risk and uncertainty, EUT. We start by identifying in general terms what is required of a procedural model of preferential choice. We then consider how the various components of such a model might be specified in ways that are in keeping with conventional economic assumptions while at the same time allowing for scarcity of time and attention. The resulting model—which we call Boundedly Rational Expected Utility Theory (BREUT)—generates a number of implications, not just about choice probabilities, but also about process measures such as response times and confidence in the decisions made.

One striking result is that, despite being based upon EU preferences, the model produces some choice patterns that deviate from EUT in line with several well-known decision-making phenomena. This highlights the influence of the processes that lead from the ‘core’ assumptions about preferences to observable choices. At the same time, there are other choice patterns that are not accommodated by BREUT. So we are not proposing BREUT as a descriptive model that can account for all known empirical regularities; nor is it intended to provide a literal representation of the way the mind actually operates. Rather, this paper may be understood as a ‘proof of concept’ exercise, which demonstrates the implications of embedding a deterministic core in a simple boundedly rational apparatus to generate decisions. As we shall explain in due course, our broad modelling strategy has the potential to be extended to many non-EU core theories, some of which may accommodate more or other known regularities.

In the next section, we present our instantiation of BREUT, focusing upon the kind of binary choices between lotteries with monetary outcomes that have been the staple diet of many decision-making experiments. In Section 3, we demonstrate how BREUT provides a parsimonious account of the systematic relationship between choice probabilities, decision time and confidence. We show that the model entails respect for first order stochastic dominance and weak stochastic transitivity, but allows patterns of choice that violate strong stochastic transitivity, independence and (to some extent) betweenness. In the final section, we consider the relationship between our model and others in the psychology and economics literature. We discuss some limitations of the model in its current form, together with what we see as the most promising directions for extending this approach. Some theorems and their proofs can be found in the online appendix.

## The model

Bounded rationality has often been characterised in terms of the difficulties DMs may encounter when they are faced with complex problems or environments involving information that is hard to obtain and compute. However, we suggest that bounded rationality may play a role even for relatively simple decisions. Even when there are just two options, the DM will need to identify and evaluate the arguments pulling in opposing directions. Because the DM cannot dwell on a decision indefinitely, she will need some mechanism to decide when to terminate her deliberation and move to a different task. It is the process underlying the allocation of time and attention between different decisions that we regard as fundamental to the characterisation of bounded rationality.

Behavioural scientists have invested substantial effort in developing various approaches for modelling decision-making processes: see, for example, elimination by aspects (Tversky 1972); the adaptive decision maker framework (Payne et al. 1993); the priority heuristic (Brandstätter et al. 2006); and query theory (Johnson et al. 2007), to name only a few. An influential stream of literature has developed an *accumulator* or *sequential-sampling* framework (for reviews, see Ratcliff and Smith 2004; Otter et al. 2008). Most such accumulator models have been developed in the context of perceptual tasks (e.g., judging the relative numbers of dots in different areas of a screen or the relative lengths of lines). The application of these models to preferential choice has been less common, although Busemeyer and Townsend’s (1993) Decision Field Theory (DFT) and its extensions constitute a notable exception. BREUT draws on this accumulator framework, and we will discuss its relationship with DFT in more detail in Section 4.

The general idea behind such models is that a DM starts without a decisive preference for any one of the available options and therefore has to acquire evidence to discriminate between them. In the case of preferential choice, this evidence comes from introspecting about the relative (subjective) desirability of the possible consequences and the weights to be attached to each of them. As evidence is accumulated, it is as if the DM continually re-assesses the relative strengths of the arguments for each option, a process that continues as long as no single option is favoured sufficiently strongly over the other(s). However, once the judged relative advantage of one option crosses some threshold, deliberation is terminated and the option favoured by the accumulated evidence is chosen. The DM can then allocate her scarce attention to something else.

Such models typically consist of three components:

(i) some representation of the sources of evidence from which samples are drawn—in this context, the underlying stock of subjective values or judgments;
(ii) some account of the way the sampled evidence is accumulated;
(iii) a stopping rule which terminates the accumulation process and triggers a decision.

In the next three subsections, we explain how BREUT models these three components. In each case, we try to make the simplest possible assumptions.

### The structure of underlying subjective values

We will be concerned with choices between pairs of lotteries of the form *L* = (*x*_1_, *p*_1_; *x*_2_, *p*_2_; …; *x*_*n*_, *p*_*n*_), where consequence *x*_*i*_ occurs with probability *p*_*i*_, ${\sum}_{i=1}^n{p}_i=1$. In deterministic EUT, DMs are assumed to choose the lottery for which ${\sum}_{i=1}^n{p}_iu\left({x}_i\right)$ is higher, where *u*(.) is a von Neumann-Morgenstern (vNM) utility function.

Let us start with the following seemingly simple decision. A DM is asked to choose between lottery *A*, which (omitting any currency symbol) offers the certainty of 30, and lottery *B*, which offers a 0.8 chance of 40 and a 0.2 chance of 0. Using standard EUT notation, with ≽ denoting weak preference, the decision can be written as:

1 $$ A\succcurlyeq B\Longleftrightarrow u(30)\kern0.5em \ge \kern0.5em 0.8\ u(40)+0.2\ u(0). $$

Deterministic EUT supposes that it is as if each DM acts according to a single utility function that gives an exact answer to this question (and gives the same answer every time that this question is presented to her). But cognitive psychology and neuroscience suggest that there is no unique and instantly accessible subjective value function—see, for example, Busemeyer and Townsend (1993), Gold and Shadlen (2007), Stewart et al. (2006, 2015). Rather, a typical DM will have many experiences and impressions of what 30 and 40 represent in subjective terms, and it may not be immediately obvious exactly where the subjective value of 30 is located in the range between the subjective values of 0 and 40, nor precisely how the differences, weighted by the probabilities, balance out. So arriving at a decision may involve deliberating about the balance of evidence obtained by sampling from those experiences and impressions.

Since we are investigating the effects of embedding EUT in a boundedly rational deliberation process, we represent the underlying stock, or *core*, of past experiences and impressions by a distribution of vNM utility functions, *u*(.), normalised so that *u*(0) = 0.

The idea that an individual’s underlying preferences might be represented by a distribution of vNM functions was suggested by Becker et al. (1963a) when they proposed their “random utility model for wagers”, which has since come to be known as the *random preference* (RP) model. The key difference between the RP model and ours is that Becker et al. supposed that DMs only sampled once for each decision, applying a single randomly-picked *u*(.) to both options, whereas we assume that deliberation involves sampling multiple times, as we now describe.

### Modelling the sampling and accumulation of evidence

Adapting the general framework of accumulator models to the specific context of binary choice between lotteries, BREUT models each sample as an independent random draw of a utility function from the core distribution of *u*(.), which is then applied to both of the lotteries under consideration. Using probabilities as weights, as EUT entails, this yields a subjective value difference which we denote by *V*(*A*, *B*), and which takes some positive value when a *u*(.) is drawn that strictly favours *A*, takes a value of zero when the two options are exactly balanced, and takes a negative value when the sampled *u*(.) strictly favours *B*. We can represent this difference as a difference between the monetary certainty equivalents (*CE*s) of the two options. Formally, for any *u*(.) sampled,

2 $$ V\left(A,B\right)={CE}_A-{CE}_B={u}^{-1}\left({EU}_A\right)-{u}^{-1}\left({EU}_B\right). $$

We use differences in *CE*s rather than in utilities because *CE*s are measures that can be legitimately compared and aggregated across utility functions. It is well known in economics that comparisons of utilities (or their aggregation) across different utility functions are theoretically questionable and lead to problematic results (e.g., Hammond 1993; Binmore 2009). For this reason, we put *V*(*A*, *B*) in a ‘common metric’ that can be legitimately aggregated. The use of *CE*s is a straightforward way of achieving that and has a number of precedents in the literature (e.g., Luce 1992; Luce et al. 1993; Cerreia-Vioglio et al. 2015).

For each sampled *u*(.), the *CE* difference provides a signal not only about which option is better, but also about how much better it is. Repeated sampling (with replacement) produces a series of independently and identically distributed realisations of *V*(*A*, *B*), which are accumulated by progressively updating their mean and sample standard deviation, denoted respectively by $\overline{V\left(A,B\right)}$ and *s*_*V*(*A*, *B*)_.

Let E[*V*(*A*, *B*)] denote the mean of the distribution of *CE* differences for the pair of lotteries {A, B} implied by the individual’s underlying population of *u*(.). If the individual takes a sample of size *k* from this distribution, that sample will have a mean which we denote by $\overline{V_k\left(A,B\right)}$. Taking sufficiently many samples of size *k* will result in some distribution for $\overline{V_k\left(A,B\right)}$. As *k* becomes larger, the distribution of $\overline{V_k\left(A,B\right)}$ will be increasingly similar to a normal distribution with a variance inversely related to *k*. If the individual were to deliberate indefinitely—that is, if *k* were allowed to tend towards infinity—the variance of the distribution of $\overline{V_k\left(A,B\right)}$ would tend towards zero and the whole distribution would collapse towards E[*V*(*A*, *B*)]. This straightforward implication of the central limit theorem will be particularly useful to shed light on the operation of our model in the following sections. In essence, if an individual had unlimited time to devote to any one decision, that would constitute a *limiting case* in which she would always arrive at the same judgment about the sign and size of $\overline{V\left(A,B\right)}$, as given by E[*V*(*A*, *B*)], with no variability. From this perspective, deterministic models may be regarded as the limiting cases in the absence of scarce time and attention. However, since unlimited deliberation is impractical, the individual will need to decide when the accumulated evidence is sufficient to make a decision. That is the role of the stopping rule.

### Modelling the stopping rule

Unlike other accumulator models in which the process of evidence accumulation terminates when an arbitrary threshold is reached, we propose an approach which sets thresholds that are responsive both to the evolving pattern of the evidence as it accumulates and to the DM’s wish to limit the time spent deliberating about any particular decision. The key to our stopping rule is the DM’s *desired level of confidence*: the DM deliberates until she concludes that the accumulated evidence gives her sufficient confidence to make a choice. In BREUT, confidence is represented as the probability that the DM picks the option that she would choose after unlimited deliberation—i.e., the option implied by the sign of E[*V*(*A*, *B*)].

We suggest that the notion of an individual attempting to achieve a personal desired level of confidence is a simple and intuitive way of building a satisficing model, and also one for which there is some empirical support (see Hausmann and Läge 2008). In contrast with the optimal stopping tradition (see, e.g., Stigler 1961; Shiryaev 1978), this approach does not require us to assume that the individual has detailed knowledge about the opportunity cost of additional sampling in terms of forgone benefits from potential future activities.

We denote the DM’s desired level of confidence as *Conf*. Because deliberation is costly in terms of the opportunity costs associated with each extra draw, we allow the DM to progressively reduce *Conf* as the amount of sampling increases. The idea here is that the longer she spends trying to discriminate between options, the more likely she is to conclude that there is not much between them, so that she has less to fear from choosing the wrong option. Specifically, we assume that the desired level of confidence after *k* draws is given by:

3 $$ Conf=\max \left[0.5,1-d\left(k-1\right)\right], $$

where *k* ≥ 2 and where *d* (with 0 < *d* ≤ 0.5) is a parameter that captures the rate at which the DM reduces her *Conf* as *k* increases, subject to the constraint that *Conf* ≥ 0.5.

*d* may vary from one individual to another, reflecting different tastes for the trade-off between more input into the current decision and turning attention to something else. A person with a very low *d* is someone who wants to be very confident in her decisions, and therefore is willing to invest more time deliberating. The limiting case is when *d* → 0, in which the individual wants to be absolutely sure of making the right decision and deliberates indefinitely. On the other hand, someone with a high value of *d* is ready to make decisions with less confidence and spends relatively less time deliberating. Thus when *d* has the maximum value of 0.5, the DM makes her decision after just two samples, choosing the option favoured by the mean of the two sampled *CE* differences. Modelling *d* as a personal characteristic provides a degree of within-person consistency with just one parameter, while allowing for heterogeneity between people. It would be possible to construct a more complicated function for *Conf*, but the linear form in Expression 3 is sufficient for our ‘proof of concept’ purposes.

We then model the DM’s decision about whether or not she should terminate her deliberation after any given sample as if she were applying a sequential *t*-test. Other ways of modelling the stopping rule are possible, but a sequential statistical test meshes well with the idea of achieving some personal level of confidence. What we try to capture is the idea that when the choice is initially presented—i.e., before any deliberation has occurred—it is as if the DM starts with the null hypothesis that there is no significant difference between the subjective values of the two options. However, as the evidence accumulates, it is as if she continually updates $\overline{V\left(A,B\right)}$ and *s*_*V*(*A*, *B*)_ and combines them to form a test statistic *T*_*k*_:

4 $$ {T}_k=\frac{\overline{V\left(A,B\right)}}{s_{V\left(A,B\right)}/\sqrt{k}}. $$

This statistic is then used to determine whether the null hypothesis of zero difference can be rejected at the level of *Conf* corresponding to *k*. This occurs if the following condition is met:

5 $$ {F}_{k-1}\left[\mathrm{abs}\left({T}_k\right)\right]\ge Conf, $$

where *F*_*k – 1*_[·] is the c.d.f. of the *t*-distribution with *k* – 1 degrees of freedom. If the weak inequality in Expression 5 is not satisfied, the DM is assumed to continue sampling and to progressively reduce *Conf* until the hypothesis of zero difference between the options is rejected in favour of one of the alternatives—at which point, she chooses whichever option is favoured by the evidence according to the sign of $\overline{V\left(A,B\right)}$. The value of *k* when sampling stops and a choice is made is denoted by k*, the level of confidence at that point is Conf* and the value of the test statistic at that point is *T*_k*_.

### Behavioural variables generated by BREUT

Because of its procedural nature, BREUT gives a richer description of decision making than process-free models, as captured by the following three variables: choice probabilities, confidence and response times. We now consider each of these in more detail.

#### Choice probabilities

In BREUT, the probability of choosing *A* over *B*, denoted by Pr(*A*≻*B*), is the probability that the null hypothesis is rejected with a positive *T*_k*_. The complementary probability that *B* is chosen is the probability that the null hypothesis is rejected with a negative *T*_k*_.

In the classic RP model, it is as if an individual samples just once per decision and chooses on the basis of the single *u*(.) sampled on that occasion. In that one-shot model, the probability of choosing *A* over *B* is given by the proportion of utility functions that favour *A* over *B* in the underlying core distribution. We denote this probability by Pr *Core*(*A*≻*B*). However, BREUT supposes that an individual samples more than once, so that the effective Pr(*A*≻*B*) will typically be different from Pr *Core*(*A*≻*B*). As noted in Section 2.2, the central limit theorem implies that the variance of the distribution of $\overline{V_k\left(A,B\right)}$ decreases and the whole distribution gets more concentrated around E[*V*(*A*, *B*)] as *k* becomes larger. As a consequence, as *k* increases, the DM becomes increasingly likely to choose the option favoured by the sign of E[*V*(*A*, *B*)]. In the limit, the probability of choosing *A* over *B* as *k* tends towards infinity, denoted by Pr *Lim*(*A*≻*B*), is either 0 (if E[*V*(*A*, *B*)] is negative) or 1 (if E[*V*(*A*, *B*)] is positive). This means that:

6 $$ {\displaystyle \begin{array}{cc}0=\Pr\ Lim\left(A\succ B\right)\le \Pr \left(A\succ B\right)\le \Pr\ Core\left(A\succ B\right)& \mathrm{if}\ E\left[V\left(A,B\right)\right]<0\\ {}\Pr\ Core\left(A\succ B\right)\le \Pr \left(A\succ B\right)\le \Pr\ Lim\left(A\succ B\right)=1& \mathrm{if}\ E\left[V\left(A,B\right)\right]>0.\end{array}} $$

That is, Pr(*A*≻*B*) always lies between the core probability in the one-shot RP model and the limiting probability (0 or 1) implied by the sign of the mean of the distribution of *CE* differences.

Expression 6 has some important implications. First, if one of the lotteries is never favoured by the individual’s core utility functions (i.e., Pr *Core*(*A*≻*B*) is either 0 or 1), then there will be no amount of sampling for which that lottery will be chosen with positive probability (i.e., it will always be the case that Pr(*A*≻*B*) = Pr *Core*(*A*≻*B*) = Pr *Lim*(*A*≻*B*)). Since dominated lotteries are never chosen in EUT, this entails that BREUT satisfies first-order stochastic dominance. Second, since (other things being equal) lower values of *d* imply larger values of k*, Pr(*A*≻*B*) will tend towards Pr *Lim*(*A*≻*B*) as *d* decreases: in other words, with more deliberation, choice probabilities become more extreme. Third, if Pr *Lim*(*A*≻*B*) and Pr *Core*(*A*≻*B*) are on different sides of 0.5 for some core, then BREUT allows for one lottery to be the modal choice even though the majority of the DM’s core utility functions favour the other lottery (see Section 3.3 and Theorems 1 and 3 in the online appendix).

#### Confidence

In BREUT, the degree of confidence in a decision, Conf*, is the level of *Conf* used in the test that rejects the null hypothesis and triggers a choice in favour of one of the alternatives after k* samples.

While a substantial literature has investigated and modelled confidence judgments in decision tasks that have a correct answer (see Pleskac and Busemeyer 2010, and references therein), there are only a few isolated exceptions of studies investigating confidence judgments in preferential choice (e.g., Butler and Loomes 1988; Sieck and Yates 1997). Whereas response times and choice probabilities are directly observable, confidence is a latent variable that is typically elicited by asking people about it, which can be done in a variety of ways (e.g., Koriat et al. 1980; Koehler 1991; Griffin and Tversky 1992). To the best of our knowledge, there are no other decision models that explicitly address confidence in preferential decisions, although confidence is clearly regarded as an important factor in various areas of economics (e.g., Acemoglu and Scott 1994; Ludvigson 2004; Dominitz and Manski 2004; Barsky and Sims 2012).^5^

#### Response time

Since deliberation is modelled through sequential sampling, BREUT can make predictions about the length of time that it takes to make a decision—the *response time* (*RT*). It is reasonable to assume that *RT* is an increasing function of the number of samples, k*, required to reject the null hypothesis. In addition, *RT* can also be expected to be positively related to the complexity of the decision problem. As there is no generally agreed index of complexity for such choices, for the purposes of this paper we make the simple assumption that complexity is reflected by the total number of consequences (*NC*) appearing in any pair.^6^ This is a straightforward way of capturing the intuition that, if there are more items to consider, each deliberation step will take longer. So we propose the following basic specification for *RT*:

7 $$ RT={k}^{\ast}\bullet NC. $$

Since k* is a stochastic variable, *RT* is also stochastic, implying that the DM is liable to take different amounts of time to reach a decision for the same pair of alternatives on different occasions. Given Expression 3 and the definition of k*, it follows that *RT* = [(1 – Conf*)/*d* + 1]*NC*. That is, *RT* and Conf* are inversely related. Also, we assume that *RT* only depends on the other parameters of the model and is not directly affected by any other contextual features. In practice, the magnitude of response times is likely to be affected by other factors, such as the particular ways in which the alternatives are displayed, the time needed to scan the stimuli, the respondent’s familiarity with the nature and format of the task, her degree of fatigue and so on. More elaborate expressions could be constructed to allow for such considerations, including a scaling factor that maps *RT*s to real time if the model is to be fitted to data, but Expression 7 is adequate for our immediate purposes.

## Exploring the predictions of BREUT

To explore the predictions of BREUT as a function of its parameters, we conducted simulations in which the utility functions are assumed to be drawn from a fixed population of power functions of the following form (some theorems for the limiting case of *d* → 0 can be found in the online appendix):

8 $$ U(x)={x}^{1-r}, $$

with *r* < 1. *r* is a random variable that varies from trial to trial, is independently and identically distributed, and can produce risk seeking (*r* < 1), risk neutrality (*r* = 0) and risk aversion (0 < *r* < 1).^7^

We assume that each time the DM samples from her core preferences, it is as if an *r* is extracted from a transformed beta distribution of risk attitude parameters, such that:

9 $$ r\sim \mathrm{Beta}\left(3,3\right)\bullet \beta +\left(\alpha -\frac{\beta }{2}\right). $$

This means that her *r* values are drawn from a symmetric and bell-shaped distribution with mean *α* and range *β*, which is bounded below and above at *α* – *β*/2 and *α + β*/2 respectively. If *β* = 0, the specification reduces to deterministic EUT with *r* = *α*.

Using this core structure, we implement the model by making independent random draws of values of *r*, each of which entails a *CE* difference between the two lotteries under consideration, and we accumulate those differences according to Expression 4 until the condition in Expression 5 is met. We simulate this process 20,000 times (unless otherwise stated) for each binary choice to generate choice probabilities, mean RTs and average Conf*.^8^

In the rest of the section, we examine how the model behaves in three respects. First, we show the results of comparing a fixed lottery to a monotonic sequence of sure alternatives when we hold the parameters of the model constant. Second, we explore how changes in each of *α*, *β* and *d* affect BREUT’s predictions when we hold the alternatives constant. Third, we use more specific sets of decision problems to illustrate how BREUT behaves in scenarios involving stochastic dominance, transitivity, independence, and betweenness.

### Fixed lottery vs. variable sure amounts

Table 1 shows how choice probabilities, confidence and response times vary in choices between a fixed lottery and a series of sure amounts of money when we set the parameters at *α* = 0.35, *β* = 1.0 and *d* = 0.1. The fixed lottery *B* (shown in the heading of the table) offers a payoff of 40 with probability 0.8 and zero with probability 0.2, represented as (**40**, 0.8; **0**, 0.2). The sure amounts *A* (shown in the first column) increase from 20 to 32 in steps of 2.

**Table 1:** Choice between a fixed lottery *B* = (**40**, 0.8; **0**, 0.2) and increasing sure amounts of money *A* (*α* = 0.35, *β* = 1.0, *d* = 0.1)

*A*	Pr *Core* (*A*≻*B*)	E[*V*(*A*, *B*)]	Pr(*A*≻*B*)	*RT*	Conf*
(**20**, 1)	0.04	−7.58	0.00	7.02	0.87
(**22**, 1)	0.08	−5.60	0.00	7.56	0.85
(**24**, 1)	0.15	−3.60	0.02	8.46	0.82
(**26**, 1)	0.26	−1.58	0.13	9.90	0.77
(**28**, 1)	0.45	0.42	0.50	10.99	0.73
(**30**, 1)	0.73	2.41	0.92	9.63	0.78
(**32**, 1)	0.97	4.41	1.00	7.83	0.84

As should be expected, increasing the value of *A* raises the proportion of utility functions favouring *A* over *B*, so that Pr *Core*(*A*≻*B*) rises from 0.04 (when *A* is 20) to 0.97 (when *A* is 32). For the values of *A* from 20 to 26 inclusive, the mean of the core distribution of *CE* differences is negative, as shown in the E[*V*(*A*, *B*)] column, so that Pr *Lim*(*A*≻*B*) = 0.^9^ In these cases, repeated sampling moves Pr(*A*≻*B*) away from Pr *Core*(*A*≻*B*) towards 0, as entailed by Expression 6. Even when 26% of *u*(.) favour *A*, as in the fourth row where *A* = 26, repeated sampling results in *A* being picked on only 13% of occasions.

However, when *A* increases to 28, E[*V*(*A*, *B*)] becomes positive, so that Pr *Lim*(*A*≻*B*) = 1 for this and all higher values of *A*. This means that for the remaining rows in the table, repeated sampling moves Pr(*A*≻*B*) above Pr *Core*(*A*≻*B*). As a consequence, even though just 45% of *u*(.) favour *A* when *A* = 28, *A* is chosen more often—in this example, in slightly more than 50% of the simulation runs, resulting in Pr(*A*≻*B*) and Pr *Core*(*A*≻*B*) being on different sides of 0.5. When *A* = 30 and is favoured by 73% of *u*(.), the deliberative process results in *A* being chosen in 92% of runs.

The next two columns show the average *RT* and the average levels of Conf*. In the rows towards the top and bottom of the table, where one or the other option is strongly favoured, *RT*s are shorter and Conf* is higher. In the middle rows, where the two options are more finely balanced, more sampling is required to reject the null hypothesis of zero difference, so that *RT*s become longer and Conf* decreases. This pattern in the *RT*s is in line with existing empirical evidence for choices between lotteries (see, e.g., Mosteller and Nogee 1951; Jamieson and Petrusic 1977; Petrusic and Jamieson 1978; Moffatt 2005).^10^ The confidence pattern is in line with the evidence found in perceptual tasks (see Pleskac and Busemeyer 2010, for a review of this literature).

### Changing the free parameters of the model

We illustrate the effect of changing *α*, *β* and *d*, in turn, by simulating choices for the pair {*A*, *B*}, with *A* = (**30**, 1) and *B* = (**40**, 0.8; **0**, 0.2).

Table 2 shows that when *α* (the mean value of *r*) is progressively increased so that the DM becomes more risk averse overall, Pr(*A*≻*B*) and Pr *Core*(*A*≻*B*) both increase monotonically, with Pr(*A*≻*B*) tending to be more extreme than Pr *Core*(*A*≻*B*), as implied by Expression 6. *RT*s tend to increase for more finely balanced decisions, while the Conf* values show the opposite pattern.

**Table 2:** Changing the median risk aversion parameter *α* (*β* = 1.0, *d* = 0.1)

*α*	Pr *Core* (*A*≻*B*)	E[*V*(*A*, *B*)]	Pr(*A*≻*B*)	*RT*	Conf*
0.05	0.20	−1.36	0.05	9.12	0.80
0.10	0.28	−0.87	0.13	10.01	0.77
0.15	0.36	−0.39	0.28	10.73	0.74
0.20	0.45	0.19	0.49	11.01	0.73
0.25	0.55	0.84	0.68	10.84	0.74
0.30	0.64	1.57	0.83	10.32	0.76
0.35	0.73	2.41	0.92	9.65	0.78
0.40	0.80	3.39	0.97	9.01	0.80

*A* = (**30**, 1)

*B* = (**40**, 0.8; **0**, 0.2)

Table 3 shows that, as *β* is increased (widening the range of *r*), more sampling is required to trigger a decision, which results in longer *RT*s and lower confidence.

**Table 3:** Changing the range of the distribution of risk aversion coefficients *β* (*α* = 0.30, *d* = 0.1)

*β*	Pr *Core* (*A*≻*B*)	E[*V*(*A*, *B*)]	Pr(*A*≻*B*)	*RT*	Conf*
0.25	0.94	0.96	1.00	7.72	0.84
0.40	0.82	1.01	0.97	8.81	0.81
0.55	0.75	1.10	0.93	9.51	0.78
0.70	0.70	1.21	0.89	9.97	0.77
0.85	0.66	1.38	0.86	10.18	0.76
1.00	0.64	1.60	0.83	10.31	0.76
1.15	0.62	1.78	0.81	10.38	0.75
1.30	0.61	2.13	0.80	10.43	0.75

*A* = (**30**, 1)

*B =* (**40**, 0.8; **0**, 0.2)

Table 4 shows the effect of decreasing *d*. When *d* = 0.5 (so that k* = 2), Pr(*A*≻*B*) goes from the Pr *Core*(*A*≻*B*) level of 0.64 to 0.75 (note that since E[*V*(*A*, *B*)] > 0, Pr *Lim*(*A*≻*B*) = 1 in this case). At the other extreme, when *d* = 0.01, Pr(*A*≻*B*) is much closer to the limiting probability of 1. Lower values of *d* mean that the DM is less willing to reduce her desired level of confidence, which entails increasing the average amount of sampling and the average *RT*s. So in the third row, where *d* = 0.3, average *RT*s are 7.01, reflecting the fact that with *NC* = 3, the average k* is 2.34; in the sixth row, where *d* = 0.1, *RT*s are around 10. The *RT*s continue to rise as *d* falls further.

**Table 4:** Changing the desired level of confidence decrease parameter *d* (*α* = 0.30, *β* = 1.0)

*d*	Pr *Core* (*A*≻*B*)	E[*V*(*A*, *B*)]	Pr(*A*≻*B*)	*RT*	Conf*
0.50	0.64	1.57	0.75	6.00	0.50
0.40	0.64	1.57	0.76	6.42	0.59
0.30	0.64	1.57	0.77	7.01	0.63
0.20	0.64	1.57	0.79	8.00	0.68
0.15	0.64	1.57	0.81	8.81	0.71
0.10	0.64	1.57	0.84	10.31	0.76
0.05	0.64	1.57	0.89	13.94	0.82
0.03	0.64	1.57	0.92	17.54	0.85
0.02	0.64	1.57	0.94	20.87	0.88
0.01	0.64	1.57	0.96	27.75	0.92

*A* = (**30**, 1)

*B* = (**40**, 0.8; **0**, 0.2)

### Implications for stochastic dominance, transitivity, independence and betweenness

We now apply BREUT to some specific problems which are typical of those used in many experimental studies. We explore the extent to which BREUT’s predictions do or do not correspond with various well-known patterns.

#### First order stochastic dominance

As noted in Section 2.4, Expression 6 entails that whenever one of the lotteries is never favoured by the core utility functions there is no amount of sampling for which that lottery will be chosen with positive probability. This condition is trivially satisfied in the case of first order stochastic dominance (FOSD). Our simulations look at the behaviour of BREUT’s procedural measures, *RT* and Conf*, for different FOSD lottery pairs.

The two pairs in Table 5 involve transparent FOSD. All lotteries offer 50–50 chances of zero or a positive payoff, with *A* offering a higher positive payoff than *B* in both pairs, 10 more in the first and 1 more in the second, so that Pr *Core*(*A*≻*B*), Pr *Lim*(*A*≻*B*) and Pr(*A*≻*B*) all equal 1. In spite of the larger payoff difference in favour of *A* in the first pair, decisions are reached quickly and with high confidence in both cases, resulting in virtually identical *RT*s and Conf*.^11^

**Table 5:** Choosing between dominating and dominated lotteries (*α* = 0.23, *β* = 1.0, d = 0.1)

*A Dominating*	*B Dominated*	Pr *Core* (*A*≻*B*)	E[*V*(*A*, *B*)]	Pr(*A*≻*B*)	*RT*	Conf*
(**60**, 0.5; **0**, 0.5)	(**50**, 0.5; **0**, 0.5)	1.00	3.50	1.00	8.36	0.89
(**51**, 0.5; **0**, 0.5	(**50**, 0.5; **0**, 0.5)	1.00	0.35	1.00	8.35	0.89

#### Weak and strong stochastic transitivity

In the probabilistic choice literature, a distinction has been made between weak stochastic transitivity (WST) and strong stochastic transitivity (SST). For any three options *X*, *Y*, *Z*, WST requires that if Pr(*X*≻*Y*) ≥ 0.5 and Pr(*Y*≻*Z*) ≥ 0.5, then Pr(*X*≻*Z*) ≥ 0.5. The stronger requirement in SST is that if Pr(*X*≻*Y*) ≥ 0.5 and Pr(*Y*≻*Z*) ≥ 0.5, then Pr(*X*≻*Z*) must be at least as large as the greater of those two: Pr(*X*≻*Z*) ≥ max[Pr(*X*≻*Y*), Pr(*Y*≻*Z*)]. As Tversky and Russo (1969) showed, SST is equivalent to an *independence between alternatives* condition, whereby Pr(*X*≻*Z*) ≥ Pr(*Y*≻*Z*) if and only if Pr(*X*≻*W*) ≥ Pr(*Y*≻*W*) for any *X*, *Y*, *Z* and *W*. That is, the relationship between the probabilities that each of two lotteries is chosen over a common alternative should not be reversed if that alternative is changed.

Rieskamp et al. (2006) concluded that the empirical evidence of violations of WST was thin, whereas there was plentiful evidence of violations of SST. In this subsection we show that BREUT is consistent with WST—i.e., the only instances of violations of WST will be due to random variation rather than to any systematic underlying tendency—but it allows systematic violations of SST of the kinds that have been documented.

For there to be any tendency to violate WST in the limit, it would be necessary to generate a case in which the mean *CE* differences for {*X*, *Y*} and {*Y*, *Z*} are positive but the mean *CE* difference for {*X*, *Z*} is negative. However, this is clearly impossible in the limiting case, as E[*V*(*X*, *Y*)] + E[*V*(*Y*, *Z*)] = E[*V*(*X*, *Z*)]. It may be possible to produce sets of *u*(.) that give violations of WST under RP’s single-sample conditions, but in BREUT such patterns will be attenuated by repeated sampling.

However, violations of SST are a different matter. This can be conveniently illustrated with an example of the so-called *Myers effect* (Myers et al. 1965), which constitutes a violation of the independence condition as specified by Tversky and Russo (1969). Table 6 shows how the probabilities of choosing between each of two lotteries *K* and *L* and a series of sure amounts (*M*) change as the sure sums are progressively increased. The independence condition applied to this case entails that any inequality between Pr(*K*≻*M*) and Pr(*L*≻*M*) should hold for all values of *M*. As Table 6 shows, this is not the case.

**Table 6:** Comparing *K* = (**180**, 0.25; **0**, 0.75) and *L* = (**40**, 0.8; **0**, 0.2) against sure amounts *M* from 25 to 33 (*α* = 0.23, *β* = 1.0, *d* = 0.1)

*M*	*K =* (**180**, 0.25; **0**, 0.75)				
	Pr *Core* (*K*≻*M*)	E[*V*(*K*, *M*)]	Pr(*K*≻*M*)	*RT*	Conf*
(**25**, 1)	0.63	4.65	0.76	10.68	0.74
(**26**, 1)	0.60	3.55	0.71	10.80	0.74
(**27**, 1)	0.57	2.51	0.66	11.01	0.73
(**28**, 1)	0.55	1.56	0.61	11.07	0.73
(**29**, 1)	0.52	0.57	0.54	11.12	0.73
(**30**, 1)	0.49	−0.43	0.48	11.10	0.73
(**31**, 1)	0.47	−1.43	0.42	11.12	0.73
(**32**, 1)	0.44	−2.40	0.35	10.97	0.73
(**33**, 1)	0.41	−3.36	0.30	10.87	0.74
*M*	*L =* (**40**, 0.8; **0**, 0.2)				
	Pr *Core* (*L*≻*M*)	E[*V*(*L*, *M*)]	Pr(*L*≻*M*)	*RT*	Conf*
(**25**, 1)	0.94	4.43	1.00	7.36	0.85
(**26**, 1)	0.90	3.45	0.99	7.86	0.84
(**27**, 1)	0.84	2.44	0.97	8.66	0.81
(**28**, 1)	0.76	1.44	0.91	9.68	0.78
(**29**, 1)	0.64	0.44	0.71	10.71	0.74
(**30**, 1)	0.49	−0.56	0.39	10.95	0.74
(**31**, 1)	0.31	−1.56	0.11	9.93	0.77
(**32**, 1)	0.13	−2.56	0.01	8.45	0.82
(**33**, 1)	0.01	−3.56	0.00	7.66	0.84

Because *K* has a wider range of payoffs than *L*, Pr *Core*(*K*≻*M*) in the top panel of Table 6 changes more slowly than Pr *Core*(*L*≻*M*) in the bottom panel as the sure amount *M* is increased. For values of *M* below 30, Pr *Core*(*K*≻*M*) < Pr *Core*(*L*≻*M*), while the opposite is true for *M* above 30.

The patterns of *RT* and Conf* are again closely related to the patterns in choice probabilities. In the top panel, choice probabilities vary less over the range of *M* that we consider, and *RT* and Conf* also display limited variation. There is more variation in choice probabilities in the bottom panel, matched by more pronounced increases and decreases in *RT*s and the corresponding opposite patterns in Conf*.

#### Implications for independence and betweenness

**Fig. 1:**
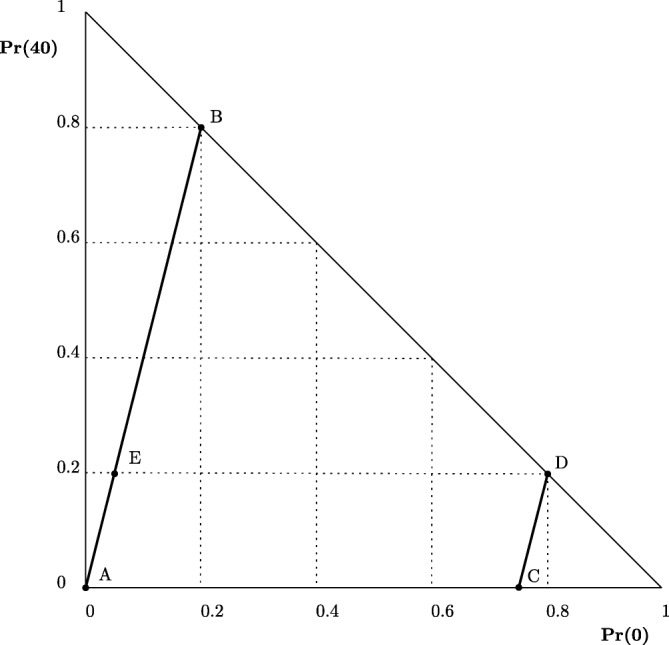
Lotteries in the Marschak-Machina triangle

Many experimental tests of the independence axiom of deterministic EUT have used pairs of lotteries that can be represented in Marschak-Machina (M-M) diagrams such as that shown in Fig. 1 (Marschak 1950; Machina 1982). For any three distinct money payoffs, *x*_*h*_ > *x*_*m*_ > *x*_*l*_ ≥ 0, the vertical axis shows the probability of receiving *x*_*h*_ and the horizontal axis shows the probability of being paid *x*_*l*_, with any residual probability assigned to *x*_*m*_. Figure 1 has been drawn for *x*_*h*_ = 40, *x*_*m*_ = 30 and *x*_*l*_ = 0, with *A* = (**30**, 1), *B* = (**40**, 0.8; **0**, 0.2), *C* = (**30**, 0.25; **0**, 0.75), *D* = (**40**, 0.2; **0**, 0.8) and *E* = (**40**, 0.2; **30**, 0.75; **0**, 0.05).

In any such triangle, deterministic EUT entails that a DM’s preferences can be represented by a set of linear and parallel indifference curves sloping up from south-west to north-east, with the gradient of the lines reflecting her attitude to risk (see Machina 1982). The straight and parallel nature of the indifference curves entails that an EU maximiser who chooses *A* (respectively, *B*) from pair {*A*, *B*} in Fig. 1, or *A* (*E*) from pair {*A*, *E*}, would also choose *C* (*D*) from pair {*C*, *D*}. This is an implication of EUT’s independence axiom. In addition, the fact that the indifference curves are linear implies that an EU maximiser choosing *A* (respectively, *B*) from pair {*A*, *B*}, would also choose *A* (*E*) from pair {*A*, *E*} and *E* (*B*) from pair {*E*, *B*}. This property is known as *betweenness*, because the intermediate lottery *E* must lie between the other two in a preference ordering.

When applied to the lotteries within an M-M triangle, Becker et al.’s RP form of EUT implies that any pair of lotteries along any straight line of a certain gradient entails the same probability of choosing the safer option in the pair (*S*) over the riskier option (*R*), with that probability, Pr *Core*(*S*≻*R*), reflecting the proportion of the DM’s vNM functions favouring *S*.^12^

Experimental work dating back to Allais (1953), Kahneman and Tversky (1979), and many others has shown that these predictions are often systematically violated. While many people are likely to choose the safer option *A* in pairs such as {*A*, *B*} and *{A*, *E*}, they tend to choose the riskier option *D* in pairs such as {*C*, *D*} much more frequently, to an extent that the modal choice often reverses. This pattern between pairs {*A*, *B*} and {*C*, *D*} has come to be known as the *common ratio* (CR) effect, while significant changes in choice frequencies between {*A*, *E*} and {*C*, *D*} constitute the *common consequence* (CC) effect. The betweenness property has also often been found to be violated, although here the experimental evidence is more variable, sometimes showing the intermediate lottery being preferred over each of the two lotteries, other times showing the opposite asymmetry (e.g., Becker et al. 1963b; Coombs and Huang 1976; Bernasconi 1994; see Blavatskyy 2006 for a recent overview).

The simulations reported in Table 7 explore the implications of BREUT for the four lottery pairs shown in Fig. 1, using three values of *d* (0.1, 0.05 and 0.01), and setting α = 0.23, *β* = 1.0. These parameter values have been chosen to obtain values of Pr *Core*(*S*≻*R*) close to 0.5, allowing us to illustrate how modal preferences can reverse. However, to show that our results are not specific to some special set of parameter values, the online appendix provides three theorems setting out general conditions under which CR, CC and betweenness effects occur.

**Table 7:** BREUT’s predictions for the independence and betweenness pairs of Fig. 1 (α = 0.23, *β* = 1.0)

*d*	Safer (*S*)	Riskier (*R*)	Pr *Core* (*S*≻*R*)	E[*V*(*S*, *R*)]	Pr(*S*≻*R*)	*RT*	Conf*
0.10	*A*	*B*	0.51	0.55	0.61	10.97	0.72
	*C*	*D*	0.51	−0.07	0.41	14.42	0.76
	*A*	*E*	0.51	0.15	0.62	14.64	0.72
	*E*	*B*	0.51	0.41	0.61	18.33	0.72
0.05	*A*	*B*	0.51	0.55	0.65	15.94	0.78
	*C*	*D*	0.51	−0.07	0.37	20.86	0.82
	*A*	*E*	0.51	0.15	0.65	21.18	0.78
	*E*	*B*	0.51	0.41	0.65	26.71	0.77
0.01	*A*	*B*	0.51	0.55	0.80	44.34	0.85
	*C*	*D*	0.51	−0.07	0.23	59.91	0.91
	*A*	*E*	0.51	0.15	0.81	58.31	0.86
	*E*	*B*	0.51	0.41	0.80	73.92	0.85

*A* = (**30**, 1)

*B* = (**40**, 0.8; **0**, 0.2)

*C* = (**30**, 0.25; **0**, 0.75)

*D* = (**40**, 0.2; **0**, 0.8)

*E* = (**40**, 0.2; **30**, 0.75; **0**, 0.05)

We start by looking at the E[*V*(*S*, *R*)] column of Table 7. For all pairs except {*C*, *D*}, E[*V*(*S*, *R*)] is positive, so we can expect that in each of these pairs the safer option will be chosen with probability 1 with unlimited sampling. The negative value for {*C*, *D*} implies that in the limit the riskier option will be chosen with probability 1, in line with both the CR and CC effects. Because of the signs of E[*V*(*S*, *R*)] and the fact that Pr *Core*(*S*≻*R*) = 0.51, Expression 6 implies that, with limited sampling, there will be a reversal of modal choice in both the CR and CC scenarios, as we see in all panels of Table 7. As *d* decreases and the DM samples more, Pr(*S*≻*R*) moves further away from 0.5, *RT*s increase on average and the DM makes her choices with higher confidence.

However, whereas BREUT readily produces CR and CC effects, the conditions under which it generates violations of betweenness are much more limited (see Theorem 2 in the online appendix for details) and in Table 7 there are no violations of probabilistic betweenness. To the extent that there are substantial systematic violations of betweenness reported in the empirical literature, BREUT does not provide a good account of these.

## Discussion and conclusions

In this final section, we consider where BREUT stands in relation to various other models, discuss some of its limitations and identify possible extensions, expand on some broader implications of our findings and offer some concluding remarks.

### BREUT vs. other stochastic models of risky choice

BREUT is quite different in terms of the structure and implications from what is arguably the most standard way of incorporating stochasticity into choice under risk: the Fechnerian version of EUT (e.g., Fechner 1860/1966; Hey and Orme 1994). In Fechnerian models, as applied by economists to EUT, a DM is assumed to have a single vNM utility function which gives a ‘true’ utility difference for one option over another, but her choices are made stochastic by adding some extraneous noise or ‘error’ component. Sometimes this will reinforce her true preference, but sometimes it may work in the opposite direction and—if sufficiently large—may outweigh the true difference, so that on some occasions the DM chooses the truly-less-preferred option. Moreover, the frequency with which this occurs may vary from pair to pair, depending on the relationship between the true utility difference and the variance of the error term.

Homoscedastic Fechnerian noise may produce patterns resembling the CR effect, because the utility difference in the {*C*, *D*} pair is smaller than in the {*A*, *B*} pair. However, with a zero-median error term, this would not generate the reversal of modal choice that is so often observed. Moreover, because the *EU* differences for pairs such as {*A*, *E*} and {*C*, *D*} are of the same magnitude, the model in its simple standard form has no way of producing the CC effect. As shown in Section 3.3, for both the CR and CC effects, BREUT’s implications are clearly different. In addition, the Fechner model would in some cases lead us to expect many more violations of FOSD than are typically observed when dominance is transparent and easy to detect (see Bardsley et al. 2010, Chapter 7, for a discussion).

Some recent refinements of the standard Fechnerian approach have tried to address some of these issues (e.g., Blavatskyy 2007, 2011; Wilcox 2011). However, these more elaborate versions offer no account for the differences in response times and confidence which, as we have seen, are intrinsic to a deliberative process such as the one modelled in BREUT and also consistent with empirical evidence. So, while we do not deny that extraneous noise may have some effect upon the choices we observe, our analysis does not depend on it and all our results are derived as if there were no such additional component.

The model that bears the closest resemblance to BREUT in the decision-making literature is one of the intermediate stages considered by Busemeyer and Townsend (1993) in their derivation of Decision Field Theory (DFT). In that paper, DFT is presented as the culmination of seven consecutive stages of modelling, running from deterministic Subjective EUT (Stage 1) to the fully-fledged version of the DFT model (Stage 7). Stage 2 modifies standard Subjective EUT by introducing fluctuating attention weights and Stage 3 embeds this model in a sequential-sampling framework. At first glance it might seem that Stage 3 DFT is much the same as the model we are proposing, but there are four key differences.

First, in Stage 3 DFT, the specification assumes that the stochastic variability is produced by fluctuations in the probabilities of comparing different pairs of payoffs, rather than fluctuations in the subjective values of those payoffs, which is what BREUT focuses upon. Second, the probabilities are transformed in a way that deviates from EUT. This alone is sufficient to produce non-EU patterns of choice and makes the model unsuitable for exploring the consequences of deliberation based strictly upon EU preferences (which is one of the main goals of this paper). Third, the magnitudes being accumulated in Stage 3 DFT are not differences in *CE*s, but differences between the utilities of the alternative options after they have been weighted by transformed probabilities. Fourth, the sequential-sampling process in DFT takes the form of a Markov process with absorbing thresholds, whereas BREUT uses a stopping rule based on a sequential statistical test. This allows BREUT to make predictions about confidence, which standard DFT (either Stage 3 or the fully-fledged version) is silent about.

After Stage 3, there are various additional stages of elaboration, so that the full (Stage 7) DFT model is considerably more complex than BREUT and involves a larger number of parameters. As a result, this full form of DFT can explain some aspects of decision behaviour that BREUT cannot, such as violations of FOSD for gambles with negatively correlated consequences (see Busemeyer and Townsend 1993, p. 447).

### Possible extensions

As stated in the introduction, our objective has not been to reconcile all of the main regularities in risky choice experiments with an EUT core, but rather to explore the implications of embedding a standard deterministic core in a boundedly rational deliberation process. As we indicated, our general modelling approach is not specific to EUT but can be extended to any other core theory that is able to generate *CE* differences between two options, which makes it potentially very widely applicable.

Of course, tracing the implications of embedding each of the very many non-EU core theories in a sequential-sampling framework would require a substantial programme of research that goes beyond the scope of this paper. However, doing so could accommodate a broader range of regularities than BREUT can explain. For example, one way of accounting for more pronounced betweenness violations would be to include some form of probability weighting in the model. In addition, while our approach focused on the domain of gains, it would in principle be possible to also study behaviour in the domain of losses or for mixed lotteries. This might involve incorporating a reference point and allowing for asymmetric attitudes to gains and losses. In short, another candidate core model might be cumulative prospect theory (CPT, Tversky and Kahneman 1992). Such an extension would present extra challenges, since it might require sampling over more than one dimension—e.g., risk aversion and degree of probability weighting, or risk aversion and loss aversion—and modelling the joint distribution of these parameters. And even then, it would struggle to explain phenomena such as ‘event-splitting’ (Starmer and Sugden 1993; Humphrey 1995) or ‘branch-splitting’ (Birnbaum 2004) effects, which occur when a particular possible outcome of a lottery appears more than once in its description, as well as other so-called paradoxes that have been shown to refute CPT (see Birnbaum 2008).

Our focus in this paper has been upon choices between pairs of risky options, but there have also been many studies reporting anomalies when comparing binary choices with other methods of eliciting preferences. So another direction in which the modelling of deliberation could usefully be developed concerns the process by which individuals generate *matching* or *equivalence* responses. In this context, a task widely used in experiments and surveys is the request to respondents to provide a best estimate of their willingness-to-pay (WTP) or willingness-to-accept (WTA) valuations. In some studies, these are elicited using *multiple price lists*, which may be regarded as a sequence of pairwise choices. However, asking DMs to work through an ordered list is likely to entail that the choices within the list are not treated independently. Extending sequential sampling models to such tasks would therefore require us to address how this non-independence should be modelled, and what the possible implications might be. In other studies, the elicitation of WTP or WTA is done via *open-ended* questions, which require the participant to generate a single monetary value as a response. One way of adapting our model to produce responses to this latter procedure might involve sampling utility functions and accumulating the resulting *CE*s, until an appropriate stopping rule prompts the individual to state a valuation based on the information acquired up to that point. However, there may be other ways of modelling the process behind equivalence judgments,^13^ the exploration of which is a challenge that we leave for future research.

### Concluding remarks

Our analysis shows that deliberative processes may affect the extent to which observed choice probabilities reflect a DM’s underlying distribution of preferences. In BREUT, the DM’s core preferences consist exclusively of vNM utility functions, and in every computation probabilities are applied untransformed, but nevertheless the process of deliberation produces patterns of choice probabilities that systematically depart from those implied by the axioms of EUT.

Our modelling strategy is akin to multiple-selves models (e.g., Elster 1987; Alós-Ferrer and Strack 2014) in that an individual’s eventual decision may be thought to reflect the aggregate of a sample of certainty equivalents expressed by different inner selves. There is an analogy here with cases in welfare economics where public policy is based on aggregating values over a population of different individuals with heterogeneous preferences. For example, suppose one were interested in valuing different degrees of risk reduction provided by two alternative interventions using measures of WTP averaged over a sample of individuals. A between-person analogue of our key result (see also Theorem 1 in the online appendix) would suggest that, even if every person in the sample were a deterministic EU maximiser, there could be a reversal of the aggregate preference over the two interventions simply based on whether the probabilities involved were scaled up or down, even though none of the individual preferences in the population implied such a reversal.

Our results also have implications for experimental investigations of decision making. Recognising the probabilistic nature of choice, one experimental design strategy has involved trying to collect repeated responses to the same decision problems to estimate choice variability. This is a methodology that can lead to complications (e.g., fatigue, boredom, remembering previous decisions, etc.), especially if the number of repetitions is high. However, because response times and confidence judgments are systematically related to choice probabilities, investigators may be able to use *RT*s and confidence measures in conjunction with fewer repetitions to produce data sets that are less vulnerable to such complications. That implication is not specific to an EUT core, so *RT*s may therefore be a useful adjunct to many studies testing a variety of core theories.

To conclude, the BREUT model set out in this paper can be seen as an illustration of the importance of taking the deliberation process into account when modelling decisions, as advocated by Simon many years ago. Allocating theoretical and empirical effort towards exploring the ‘production of decisions’ is, we suggest, likely to open fruitful avenues for future research.

## Electronic supplementary material

ESM 1 (PDF 459 kb)

## Footnotes

1 This was the year in which Simon was awarded the Nobel Prize in economic sciences “for his pioneering research into the decision-making process within economic organizations” (www.nobelprize.org/nobel_prizes/economic-sciences/laureates/1978/simon-bio.html), and this lecture drew on ideas he had been developing since the 1950s.

2 When participants are presented with exactly the same decision task displayed in exactly the same way on two or more separate occasions within the same experiment, they often give different responses on different occasions. See Mosteller and Nogee (1951) for an early example; Luce and Suppes (1965) for a review of the early theoretical literature; and Rieskamp et al. (2006), Bardsley et al. (2010, Chapter 7), and Baucells and Villasís (2010) for more recent discussions.

3 See, for example, Tyebjee (1979), Birnbaum and Jou (1990), Busemeyer and Townsend (1993), Wilcox (1993), Moffatt (2005), Rubinstein (2007, 2013), Spiliopoulos and Ortmann (2016), Achtziger and Alós-Ferrer (2014).

4 See, for example, Butler and Loomes (1988), Sieck and Yates (1997), Pleskac and Busemeyer (2010).

5 There have been papers discussing the relationship between response time and *strength of preference* (e.g., Birnbaum and Jou 1990). However, confidence is conceptually different from strength of preference, although the two may be highly correlated in some cases.

6 If the same consequence appears in both alternatives, it is counted twice in *NC*. If it appears twice in the same alternative, it is counted once.

7 We also conducted a variety of simulations using an underlying distribution of CARA utility functions of the form *U*(*x*) = (1 – e^–*rx*^)/*r*, and also simulations restricting all functions to be concave, the results of which are available from the authors upon request.

8 All simulations and calculations were implemented using R. The code is available from the authors on request.

9 E[*V*(*A*, *B*)] has been calculated in all cases as the mean of 100,000 simulated *CE* differences.

10 The overall distribution of *RT*s tends to be positively skewed. This is often observed in experiments (e.g., Ratcliff and Smith 2004), even though this evidence typically comes from tasks in which there is a correct answer, as opposed to preferential choice.

11 Empirically, even when FOSD is transparent, there are occasional violations, and there is no discernible difference in this respect between pairs in which the increment is larger or smaller (see Butler et al. 2014a, b). One possibility would be to ascribe these to pure mistakes (e.g., simple lapses of concentration) and capture them by a *tremble* term, as in Moffatt and Peters (2001) or Loomes et al. (2002). Substantial rates of violation that cannot be ascribed to such factors (e.g., the violations in Birnbaum and Navarrete 1998, which Birnbaum 2008 has explained using his TAX model) could be interpreted as evidence against the assumption of an EUT core.

12 In each pair, the safer option is the one more to the south-west, the riskier the one more to the north-east. So, for example, *A* is the safer in {*A*, *B*} and {*A*, *E*}, *C* is the safer in {*C*, *D*}. Throughout the rest of this section, we will use the convention that the first lottery in any pair {*S*, *R*} is the safer and the second is the riskier.

13 Within the framework of DFT, Johnson and Busemeyer (2005, pp. 844–6) outlined a ‘sequential value-matching’ algorithm that represented valuation as a somewhat indirect process, a form of iterative binary choice between the option being valued and a series of possible sure amounts.
