# Supplementary material for: Boundedly rational expected utility theory
Source: J Risk Uncertain. 2018 Dec 28;57(3):199–223. doi: 10.1007/s11166-018-9293-3 (PMC6383980; doi:10.1007/s11166-018-9293-3)
Supplement: Supplementary file 1 — (PDF 459 kb) [file 11166_2018_9293_MOESM1_ESM.pdf]

Boundedly Rational Expected Utility Theory

Online Appendix

Daniel Navarro-Martinez[1], Graham Loomes[2], Andrea Isoni[2], David Butler[3], and Larbi Alaoui[1]

[1]*Department of Economics and Business, Pompeu Fabra University, and Barcelona Graduate School of Economics, Ramon Trias Fargas 25-27, 08005 Barcelona, Spain.*

[2]*Warwick Business School, University of Warwick, Coventry CV4 7AL, UK.*

[3]*Department of Accounting, Finance and Economics, Griffith Business School, Griffith University, Gold Coast, Queensland, Australia.*

Journal of Risk and Uncertainty 57(3), December 2018

This appendix contains three theorems relating to the limiting case of $d \rightarrow 0$, and their corresponding proofs, that establish general conditions under which BREUT will produce violations of independence and betweenness like the ones shown in Section 3. Theorem 1 applies the limiting case to analyse what happens when the probabilities of the best consequence are scaled down, as in the CR scenario. Theorem 2 derives more general properties about the shape of the indifference curves for cores of utility functions restricted to be either weakly concave or weakly convex, which has implications for whether or not BREUT satisfies betweenness. By combining insights from Theorem 1 and Theorem 2, Theorem 3 elaborates further on the circumstances under which the CC effect can be obtained. Theorems 1 and 2 and their proofs are followed by intuitive explanations of their implications.

The proofs below make use of the following observation. For any two lotteries A and B, if $\Pr(A \succ B) \rightarrow 1$ as $d \rightarrow 0$, then $E[V(A, B)] > 0$, and so the expected CE of A is greater than the expected CE of B (i.e., $E(CE_A) > E(CE_B)$, where, for any lottery L, $E(CE_L)$ denotes its expected CE). If $\Pr(A \succ B) \rightarrow 0.5$ as $d \rightarrow 0$, then $E[V(A, B)] = 0$ and $E(CE_A) = E(CE_B)$. Recall also that the mixture operator is defined in the usual way, i.e., if $A(x)$ and $B(x)$ denote the probability of reaching prize x in lotteries A and B, respectively, then $(\omega A + (1-\omega)B)(x) = \omega A(x) + (1-\omega)B(x)$ for all x.

Theorem 1

Let the core consist of $N > 1$ CRRA functions, i.e., functions of form $U_i(x) = x^{1-r_i}$, where $r_i < 1$ for all $i \in \{1, ..., N\}$, and assume that $r_i \neq r_j$ for at least some $i, j \in \{1, ..., N\}$. Take any lotteries $S = (x_m, p; 0, 1-p)$ and $R = (x_h, q; 0, 1-q)$ for which $x_h > x_m > 0$, $p \in (0, 1]$, $q \in (0, 1)$ and for which $\Pr(S \succ R) \rightarrow 0.5$ as $d \rightarrow 0$. Then, for any lottery $S' = (x_m, \sigma p; 0, 1-\sigma p)$ and $R' = (x_h, \sigma q; 0, 1-\sigma q)$ where $\sigma \in (0, 1)$, it must be that $\Pr(S' \succ R') \rightarrow 0$ as $d \rightarrow 0$.

Proof. For convenience, define $\theta_i = \frac{1}{1-r_i}$, so that $U_i(x) = x^{1/\theta_i}$, with $\theta_i > 0$. Without loss of generality, assume the θ_i's are ordered according to $\theta_1 > \cdots > \theta_N$. (Note that, while in principle it may be that $\theta_i = \theta_j$ for some distinct i, j, there are at least one i and one j such that $\theta_i \neq \theta_j$. So, the above ordering can be done by replacing N with the cardinality of the strict ordering, which is of at least 2.) Let f_i denote the probability of θ_i occuring. We proceed by contradiction. Suppose that the result does not hold, so that $\Pr(S \succ R) \rightarrow 0.5$ and $\Pr(S' \succ R')$ does not tend to 0 as $d \rightarrow 0$. First, $E(CE_S) = E(CE_R)$ and hence

$$x_m \sum_{i=1}^{N} f_i p^{\theta_i} = x_h \sum_{i=1}^{N} f_i q^{\theta_i}, \text{ which implies } x_m = \frac{x_h \sum_{i=1}^{N} f_i q^{\theta_i}}{\sum_{i=1}^{N} f_i p^{\theta_i}}.$$

Second, since it must be that $\Pr(S' \succ R')$ tends to a number greater than 0 as $d \rightarrow 0$ (and specifically 0.5 or 1), then $E(CE_{S'}) \geq E(CE_{R'})$, and hence

$x_m \sum_{i=1}^{N} f_i (\sigma p)^{\theta_i} \geq \; x_h \sum_{i=1}^{N} f_i (\sigma q)^{\theta_i}$, which implies $x_m \geq \frac{x_h \sum_{i=1}^{N} f_i (\sigma q)^{\theta_i}}{\sum_{i=1}^{N} f_i (\sigma p)^{\theta_i}}$.

Combining the two, we obtain:

$$\frac{\sum_{i=1}^{N} f_i q^{\theta_i}}{\sum_{i=1}^{N} f_i p^{\theta_i}} \geq \frac{\sum_{i=1}^{N} f_i (\sigma q)^{\theta_i}}{\sum_{i=1}^{N} f_i (\sigma p)^{\theta_i}},$$

where we have divided by x_h on both sides. Noting that $q < p$, we write $q = bp$, where $b = q/p \in (0, 1)$. We therefore have:

$$\frac{\sum_{i=1}^{N} f_i (bp)^{\theta_i}}{\sum_{i=1}^{N} f_i p^{\theta_i}} \geq \frac{\sum_{i=1}^{N} f_i (\sigma bp)^{\theta_i}}{\sum_{i=1}^{N} f_i (\sigma p)^{\theta_i}},$$

$$\Rightarrow \quad \sum_{j=1}^{N} f_j (bp)^{\theta_j} \sum_{i=1}^{N} f_i (\sigma p)^{\theta_i} \; \geq \; \sum_{j=1}^{N} f_j (\sigma bp)^{\theta_j} \sum_{i=1}^{N} f_i p^{\theta_i}.$$

So:

$$\left(f_1 b^{\theta_1} p^{\theta_1} + \; \ldots + \; f_N b^{\theta_N} p^{\theta_N}\right)\left(f_1 \sigma^{\theta_1} p^{\theta_1} + \; \ldots \; + \; f_N \sigma^{\theta_N} p^{\theta_N}\right) \geq$$
$$\left(f_1 \sigma^{\theta_1} b^{\theta_1} p^{\theta_1} + \; \ldots + \; f_N \sigma^{\theta_N} b^{\theta_N} p^{\theta_N}\right)\left(f_1 p^{\theta_1} + \; \ldots \; + \; f_N p^{\theta_N}\right).$$

Factoring out the left-hand side (LHS) and the right-hand side (RHS) of the equation:

$$f_1^2 b^{\theta_1} \sigma^{\theta_1} p^{2\theta_1} + \; f_1 f_2 b^{\theta_1} \sigma^{\theta_2} p^{\theta_1+\theta_2} + \; \ldots \; + \; f_1 f_N b^{\theta_1} \sigma^{\theta_N} p^{\theta_1+\theta_N} +$$
$$f_2 f_1 b^{\theta_2} \sigma^{\theta_1} p^{\theta_1+\theta_2} + \; f_2^2 b^{\theta_2} \sigma^{\theta_2} p^{2\theta_2} + \; \ldots \; + \; f_2 f_N b^{\theta_2} \sigma^{\theta_N} p^{\theta_2+\theta_N} \; +$$
$$+ \qquad \ldots \qquad\qquad\qquad +$$
$$f_N f_1 b^{\theta_N} \sigma^{\theta_1} p^{\theta_1+\theta_N} + \; f_N f_2 b^{\theta_N} \sigma^{\theta_2} p^{\theta_2+\theta_N} + \; \ldots \; + \; f_N^2 b^{\theta_N} \sigma^{\theta_N} p^{2\theta_N}$$
$$\geq$$
$$f_1^2 b^{\theta_1} \sigma^{\theta_1} p^{2\theta_1} + f_1 f_2 b^{\theta_1} \sigma^{\theta_1} p^{\theta_1+\theta_2} + \cdots + \; f_1 f_N b^{\theta_1} \sigma^{\theta_1} p^{\theta_1+\theta_N} +$$
$$f_2 f_1 b^{\theta_2} \sigma^{\theta_2} p^{\theta_1+\theta_2} + f_2^2 b^{\theta_2} \sigma^{\theta_2} p^{2\theta_2} + \cdots + \; f_2 f_N b^{\theta_2} \sigma^{\theta_2} p^{\theta_2+\theta_N} \; +$$
$$+ \qquad \ldots \qquad\qquad\qquad +$$
$$f_N f_1 b^{\theta_N} \sigma^{\theta_N} p^{\theta_1+\theta_N} + f_N f_2 b^{\theta_N} \sigma^{\theta_N} p^{\theta_2+\theta_N} + \; \ldots + \; f_N^2 b^{\theta_N} \sigma^{\theta_N} p^{2\theta_N}.$$

Canceling out the common terms on the LHS and the RHS side and grouping the terms that include $f_i f_j$ with those that include $f_j f_i$, we obtain:

$$f_1 f_2 p^{\theta_1+\theta_2}\left(b^{\theta_1}\sigma^{\theta_2} + b^{\theta_2}\sigma^{\theta_1}\right) + f_1 f_3 p^{\theta_1+\theta_3}\left(b^{\theta_1}\sigma^{\theta_3} + b^{\theta_3}\sigma^{\theta_1}\right) + \ldots + f_1 f_N p^{\theta_1+\theta_N}\left(b^{\theta_1}\sigma^{\theta_N} +\right.$$
$$\left. b^{\theta_N}\sigma^{\theta_1}\right) + f_2 f_3 p^{\theta_2+\theta_3}\left(b^{\theta_2}\sigma^{\theta_3} + b^{\theta_3}\sigma^{\theta_2}\right) + \; \ldots +$$
$$f_{N-1} f_N p^{\theta_{N-1}+\theta_N}\left(b^{\theta_{N-1}}\sigma^{\theta_N} + b^{\theta_N}\sigma^{\theta_{N-1}}\right) \quad \geq \; f_1 f_2 p^{\theta_1+\theta_2}\left(b^{\theta_1}\sigma^{\theta_1} + b^{\theta_2}\sigma^{\theta_2}\right) + f_1 f_3 p^{\theta_1+\theta_3}\left(b^{\theta_1}\sigma^{\theta_1} +\right.$$
$$\left. b^{\theta_3}\sigma^{\theta_3}\right) + f_2 f_3 p^{\theta_2+\theta_3}\left(b^{\theta_2}\sigma^{\theta_2} + b^{\theta_3}\sigma^{\theta_3}\right) + \ldots + f_{N-1} f_N p^{\theta_{N-1}+\theta_N}\left(b^{\theta_{N-1}}\sigma^{\theta_{N-1}} + b^{\theta_N}\sigma^{\theta_N}\right),$$

which can be written as:

$$\sum_{j=i+1}^{N}\sum_{i=1}^{N-1} f_i f_j p^{\theta_i+\theta_j}\left(b^{\theta_i}\sigma^{\theta_j} + b^{\theta_j}\sigma^{\theta_i}\right) \geq \sum_{j=i+1}^{N}\sum_{i=1}^{N-1} f_i f_j p^{\theta_i+\theta_j}\left(b^{\theta_i}\sigma^{\theta_i} + b^{\theta_j}\sigma^{\theta_j}\right).$$

But notice that for any $j > i$, $f_i f_j p^{\theta_i+\theta_j}\left(b^{\theta_i}\sigma^{\theta_j} + b^{\theta_j}\sigma^{\theta_i}\right) < f_i f_j p^{\theta_i+\theta_j}\left(b^{\theta_i}\sigma^{\theta_i} + b^{\theta_j}\sigma^{\theta_j}\right)$, as it is implied by $b^{\theta_i}\sigma^{\theta_j} + b^{\theta_j}\sigma^{\theta_i} < b^{\theta_i}\sigma^{\theta_i} + b^{\theta_j}\sigma^{\theta_j}$, which is implied by $b^{\theta_i}\left(\sigma^{\theta_j} - \sigma^{\theta_i}\right) < b^{\theta_j}\left(\sigma^{\theta_j} - \sigma^{\theta_i}\right)$, which is implied by $\sigma^{\theta_j} - \sigma^{\theta_i} > 0$ and by $b^{\theta_i} < b^{\theta_j}$, which are both true because $\sigma, b \in (0,1)$ and $\theta_i > \theta_j > 0$. Since this is true for each of the comparisons of the matching terms in the LHS and the RHS sums, it follows that:

$$\sum_{j=i+1}^{N}\sum_{i=1}^{N-1} f_i f_j p^{\theta_i+\theta_j}\left(b^{\theta_i}\sigma^{\theta_j} + b^{\theta_j}\sigma^{\theta_i}\right) < \sum_{j=i+1}^{N}\sum_{i=1}^{N-1} f_i f_j p^{\theta_i+\theta_j}\left(b^{\theta_i}\sigma^{\theta_i} + b^{\theta_j}\sigma^{\theta_j}\right),$$

which is a contradiction and completes the proof. ■

Theorem 1 concerns lotteries of the form typically used in CR scenarios, $S = (x_m, p; 0, 1 - p)$ and $R = (x_h, q; 0, 1 - q)$, with $x_h > x_m > 0$. The commonly used case in which $p = 1$ is also allowed by the theorem.

To illustrate the intuition, suppose that $\Pr(S \succ R) \to 0.5$ as $d \to 0$ (i.e., the decision maker is indifferent between S and R in the limit, so that $E[V(S, R)] = 0$) for some $p \leq 1$. The key result is that, for a core made of CRRA functions, scaling the probabilities of the best outcome of each lottery down by some factor σ always results in the DM having a strict preference for the scaled down risky lottery R'. Although Theorem 1 starts from perfect indifference between S and R in the scaled-up pair, an immediate corollary is that it will always be possible to obtain a strict preference in favour of S by slightly reducing q, the probability of winning x_h in R. That is, in the limit, a CR effect can always be obtained in which the DM has a strict preference for the safer lottery in the scaled-up pair and a strict preference for riskier lottery in the scaled-down pair. A preference reversal in the other direction does not occur for any lotteries of these forms.

Theorem 1 holds as long as there are at least two different CRRA utility functions in the core, without any further assumption about the core distribution, such as the degree of risk aversion implied by each function. The case of a continuous distribution (like the ones used in our simulations) can be approximated by taking an N that is sufficiently large.

Figure A.1 illustrates the implications of Theorem 1. For convenience, pairs {S, R} and {S', R'} are drawn on (dashed) lines with the same gradient as the pairs of lotteries in Figure 1, which are also shown in the Figure A.1. S is drawn for some $p < 1$ (i.e., on the bottom edge of the triangle, but away from lottery A in the bottom-left corner). Because of the requirement that the DM is indifferent

between S and R in the limit, S and R lie on the same indifference curve (the solid line connecting S and R). In the limiting case of BREUT, an indifference curve is the set of lotteries for which the core entails exactly the same mean *CE*. Theorem 1 implies that R' lies above the indifference curve passing through S'. In other words, indifference curves become flatter as one moves towards the bottom-right corner of the triangle, in line with the often discussed 'fanning out' pattern. Similarly, because the S in Figure A.1 has been selected arbitrarily, the theorem implies that, to the left of S, indifference curves will be steeper. For a DM with indifference curves like those in Figure A.1, Pr $Lim(A, B) = 1$, Pr $Lim(A, E) = 1$ and Pr $Lim(C, D) = 0$, that is, she would display both the CR and the CC patterns with probability 1 in the limit.

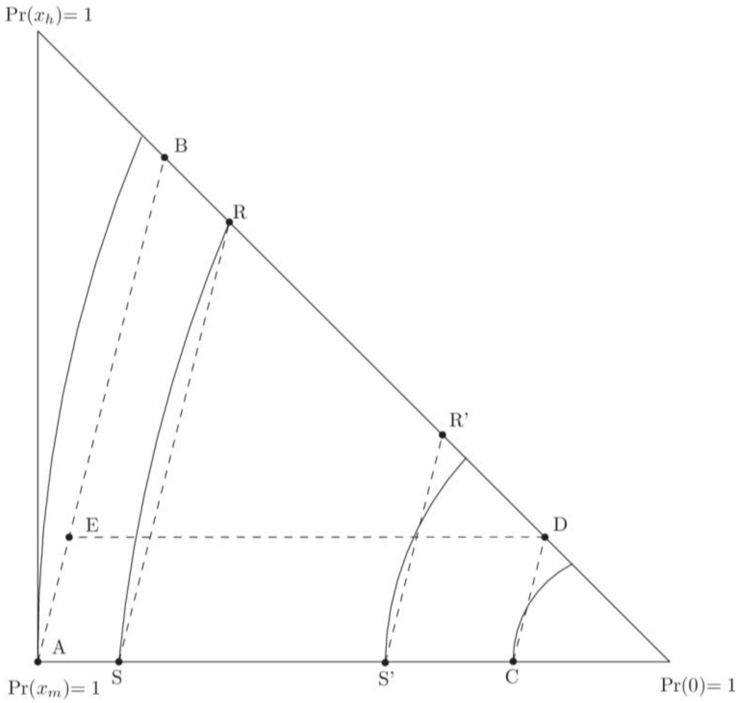

FIGURE A.1

A sketch of BREUT's indifference curves for the limiting case ($d \to 0$)

Theorem 2

Maintain the assumptions of Theorem 1 (i.e., the core consists of N CRRA functions, at least two of which are distinct). Take any distinct lotteries $S = (x_h, p_1; x_m, p_2; 0, 1- p_1 - p_2)$ and $R = (x_h, q_1; x_m, q_2; 0, 1 - q_1 - q_2)$ for which $x_h > x_m > 0$, $p_1, p_2, q_1, q_2 \in [0, 1]$, $p_1 + p_2 < 1$, $q_1 + q_2 < 1$ and for which $\Pr(S \succ R) \to 0.5$ as $d \to 0$.

Suppose that all utility functions are (weakly) concave, i.e., $0 \leq r_i < 1$ for all $i \in \{1, ..., N\}$. Then $\Pr(S \succ \omega S + (1 - \omega)R) \to 1$ and $\Pr(R \succ \omega S + (1 - \omega)R) \to 1$ as $d \to 0$, where $\omega \in (0, 1)$.

Suppose that all utility functions are (weakly) convex, i.e., $r_i \leq 0$ for all $i \in \{1, ..., N\}$. Then $\Pr(S \succ \omega S + (1 - \omega)R) \to 0$ and $\Pr(R \succ \omega S + (1 - \omega)R) \to 0$ as $d \to 0$.

Proof. We only prove the case for $0 \leq r_i < 1$ for all $i \in \{1,..., N\}$; an analogous proof holds for $r_i \leq 0$ for all $i \in \{1, ..., N\}$. Adopt the same notation as in the proof of Theorem 1, with the added restriction here that $\theta_i \geq 1$ for all i. Moreover, since there are at least two distinct $\theta's$ in the support, it must be that $\theta_i > 1$ for at least one i, i.e., it must be that at least one utility function is strictly concave.
Since $\Pr(S \succ R) \rightarrow 0.5$ as $d \rightarrow 0$, it must be that $E(CE_S) = E(CE_R)$ and hence that

$$\sum_{i=1}^{N} f_i(p_1 x_h^{1/\theta_i} + p_2 x_m^{1/\theta_i})^{\theta_i} = \sum_{i=1}^{N} f_i(q_1 x_h^{1/\theta_i} + q_2 x_m^{1/\theta_i})^{\theta_i}.$$

Moreover, since $\omega S + (1 - \omega)R = (x_h, \omega p_1 + (1 - \omega)q_1; x_m, \omega p_2 + (1 - \omega)q_2; 0, 1 - \omega (p_1 + p_2) - (1 - \omega)(q_1 + q_2))$,

$$\mathrm{E}(CE_{\omega S + (1-\omega)R}) = \sum_{i=1}^{N} f_i((\omega p_1 + (1-\omega)q_1)x_h^{1/\theta_i} + (\omega p_2 + (1-\omega)q_2)x_m^{1/\theta_i})^{\theta_i}$$

$$= \sum_{i=1}^{N} f_i(\omega(p_1 x_h^{1/\theta_i} + p_2 x_m^{1/\theta_i}) + (1-\omega)(q_1 x_h^{1/\theta_i} + q_2 x_m^{1/\theta_i}))^{\theta_i}.$$

Since $\theta_i \geq 1$ for all i and $\theta_i > 1$ for at least one i, it follows from Jensen's Inequality for weakly and strictly convex functions that:

$$(\omega(p_1 x_h^{1/\theta_i} + p_2 x_m^{1/\theta_i}) + (1-\omega)(q_1 x_h^{1/\theta_i} + q_2 x_m^{1/\theta_i}))^{\theta_i}$$
$$\leq \omega(p_1 x_h^{1/\theta_i} + p_2 x_m^{1/\theta_i})^{\theta_i} + (1-\omega)(q_1 x_h^{1/\theta_i} + q_2 x_m^{1/\theta_i})^{\theta_i}$$

for all i, with strict inequality for at least one i for which $\theta_i > 1$. The reason the inequality is strict for at least one θ_i is that equality would only hold if for all θ_i, $p_1 x_h^{1/\theta_i} + p_2 x_m^{1/\theta_i} = q_1 x_h^{1/\theta_i} + q_2 x_m^{1/\theta_i}$, i.e., $({}^{x_h}/_{x_m})^{1/\theta_i}(p_1 - q_1) = q_2 - p_2$. But this is impossible because there are at least two distinct $\theta's$ (i.e., $\theta_j \neq \theta_k$ for at least some $j, k \in \{1, ..., N\}$), and so $({}^{x_h}/_{x_m})^{1/\theta_i}$, and hence the LHS, is different for the distinct $\theta_j \neq \theta_k$, while the RHS maintains the same value, and so equality can hold at most for one θ (and note that in the case where $N = 2$, $\theta_1 > 1$, and $\theta_2 = 1$, then clearly from $E(CE_S) = E(CE_R)$, $p_1 x_h^{1/\theta_i} + p_2 x_m^{1/\theta_i} = q_1 x_h^{1/\theta_i} + q_2 x_m^{1/\theta_i}$ could not hold for either θ_1 or θ_2. Hence, even in that case, strict inequality would still hold for $\theta_1 > 1$). Therefore, combining all terms, we obtain:

$$\mathrm{E}(CE_{\omega S\,+\,(1\,-\,\omega)R}) = \sum_{i=1}^{N} f_i(\omega(p_1 x_h^{1/\theta_i} + p_2 x_m^{1/\theta_i}) + (1-\omega)\ (q_1 x_h^{1/\theta_i} + q_2 x_m^{1/\theta_i}\ ))^{\theta_i}$$

$$< \sum_{i=1}^{N} f_i(\omega(p_1 x_h^{1/\theta_i} + p_2 x_m^{1/\theta_i})^{\theta_i} + (1-\omega)(q_1 x_h^{1/\theta_i} + q_2 x_m^{1/\theta_i}\ )^{\theta_i})$$

$$= \ \omega \sum_{i=1}^{N} f_i(p_1 x_h^{1/\theta_i} + p_2 x_m^{1/\theta_i})^{\theta_i} + (1-\omega) \sum_{i=1}^{N} f_i(q_1 x_h^{1/\theta_i} + q_2 x_m^{1/\theta_i}\ )^{\theta_i}$$

$$= \omega\mathrm{E}(CE_S)\ + (1-\omega)\mathrm{E}(CE_R) = \ \mathrm{E}(CE_S) = \ \mathrm{E}(CE_R),$$

where we have used that $\mathrm{E}(CE_{\mathrm{S}}) = \sum_{i=1}^{N} f_i(p_1 x_h^{1/\theta_i} + p_2 x_m^{1/\theta_i})^{\theta_i}$ and $\mathrm{E}(CE_R) = \sum_{i=1}^{N} f_i(q_1 x_h^{1/\theta_i} + q_2 x_m^{1/\theta_i}\ )^{\theta_i}$. We have therefore shown that $\mathrm{E}(CE_{\omega S\,+\,(1\,-\,\omega)R}) < \ \mathrm{E}(CE_{\mathrm{S}}) = \mathrm{E}(CE_{\mathrm{R}})$, from which it follows that $\Pr(S \succ \omega S + (1-\omega)R) \to 1$ and $\Pr(R \succ \omega S + (1-\omega)R) \to 1$ as $d \to 0$. This concludes the proof. The proof for $r_i \leq 0$ is analogous, but uses Jensen's Inequality for concave and strictly concave functions instead. ■

According to Theorem 2, if there are only (weakly) concave utility functions in the core, BREUT's indifference curves are always concave. This is the case depicted in Figure A.1. Note that, since there must be at least two distinct utility functions in the core (as assumed in Theorem 1), the weak concavity requirement entails that there will be at least one strictly concave function. The key result is that any mixture of two lotteries, $S = (x_m, p; 0, 1-p)$ and $R = (x_h, q; 0, 1-q)$, that lie on the same indifference curve will be less preferred than either S or R. However, since the DM is assumed to be exactly indifferent between the two mixed lotteries, the degree of concavity of the indifference curves will typically be very small (unless the core is made of very extreme functions). This limits the room for observing violations of betweenness when sampling is limited (see our simulations in the main text). If the core contains only (weakly) convex utility functions, then indifference curves will be convex and any mixture of S and R will be preferred to both. If there are both concave and convex utility functions in the core, the exact shape of the indifference curves will depend on the balance between concave and convex functions and on how extreme these are.

An implication of Theorems 1 and 2 is that, while the CR effect will always be observed in the limit, for any core, the same is not guaranteed for the CC effect. But the effect will always be found in the limit if the core does not contain convex utility functions (the case illustrated in Figure A.1), as detailed in Theorem 3.

Theorem 3

Maintain the assumptions of Theorem 1 (i.e., the core consists of N CRRA functions, at least two of which are distinct). Suppose that all utility functions are (weakly) concave, i.e., $0 \leq r_i < 1$ for

all $i \in \{1, ..., N\}$. Take any lotteries $T = (x_m, 1)$ and $Z = (x_h, q_1; x_m, q_2; 0, 1 - q_1 - q_2)$ for which $x_h > x_m > 0$, $q_1, q_2 \in (0, 1)$, $q_1 + q_2 < 1$ and $\Pr(T \succ Z) \rightarrow 0.5$ as $d \rightarrow 0$. Then it must be that for $T' = (x_m, 1 - q_2; 0, q_2)$ and $Z' = (x_h, q_1; 0, 1 - q_1)$, $\Pr(T' \succ Z') \rightarrow 0$ as $d \rightarrow 0$.

Proof. Adopt the same notation as in the proofs of Theorems 1 and 2. We make use of both theorems to prove the result. First, it follows from $\Pr(T \succ Z) \rightarrow 0.5$ as $d \rightarrow 0$ that $E(CE_T) = E(CE_Z)$. Consider now the lottery $G = (x_h, \frac{q_1}{1-q_2}; 0, \frac{1-q_1-q_2}{1-q_2})$, which is at the intersection between the hypotenuse and the line segment connecting lotteries T and Z in the corresponding Marschak-Machina diagram. Note that $Z = q_2 T + (1 - q_2)G$, since $q_2 T + (1 - q_2)G = q_2(x_m, 1) + (1 - q_2)(x_h, \frac{q_1}{1-q_2}; 0, \frac{1-q_1-q_2}{1-q_2}) = (x_h, q_1; x_m, q_2; 0, 1 - q_1 - q_2)$. By Theorem 2, it cannot be that $E(CE_G) = E(CE_T)$, as this would imply that $E(CE_Z) < E(CE_T)$. It is also obvious from monotonicity that $E(CE_G)$ cannot be strictly less than $E(CE_T)$, which would also imply that $E(CE_Z) < E(CE_T)$. It must therefore be that $E(CE_G) > E(CE_T)$. Now from Theorem 1, we know that if $E(CE_T) = E(CE_G)$ holds (i.e., $\Pr(T \succ G) \rightarrow 0.5$ as $d \rightarrow 0$), then $E(CE_{T'}) < E(CE_{Z'})$ (i.e., $\Pr(T' \succ Z') \rightarrow 0$ as $d \rightarrow 0$). This can be seen by taking p in Theorem 1 to be 1, q to be $\frac{q_1}{1-q_2}$, and σ to be $1 - q_2$, and replacing S with T, R with G, S' with T' and R' with Z'. It is then clear by monotonicity that $E(CE_G) > E(CE_T)$ also implies that $\Pr(T' \succ Z') \rightarrow 0$ as $d \rightarrow 0$. ■
